# The Eustachian Tube Dysfunction in Children: Anatomical Considerations and Current Trends in Invasive Therapeutic Approaches

**DOI:** 10.7759/cureus.27193

**Published:** 2022-07-24

**Authors:** Anastasios K Goulioumis, Magioula Gkorpa, Michalis Athanasopoulos, Ioannis Athanasopoulos, Kostis Gyftopoulos

**Affiliations:** 1 Otolaryngology-Head and Neck Surgery, Karamandaneio Pediatric Hospital, Patras, GRC; 2 Anatomy, School of Medicine, University of Patras, Patras, GRC; 3 Otorhinolaryngology, Private Practice, Mesolongi, GRC; 4 Otolaryngology-Head and Neck Surgery, Pediatric Center of “Iatriko Athinon” Hospital, Athens, GRC

**Keywords:** children, balloon dilatation, otitis media with effusion, eustachian tube dysfunction, eustachian tube anatomy

## Abstract

The eustachian tube (ET) has a crucial role in the physiology of the middle ear. Thus, any condition that renders the tube dysfunctional is directly implicated with middle ear pathophysiology, like in the case of acute otitis media and otitis media with effusion. Children are more vulnerable to pathologies of the middle ear, primarily due to the immature development of their eustachian tubes. Otitis media with effusion, apart from being a burden for hearing, with direct consequences for speech development, may also be implicated in cholesteatoma formation. Medical therapy is not practically effective for the treatment of effusion. Moreover, the established surgical approaches, like grommets and adenoidectomy, deal only indirectly with the problem since they are not addressing the dysfunctional ET itself. An emerging interventional approach that intends to restore the function of the tube is the transnasal balloon dilation of the cartilaginous part of the ET. Growing international experience indicates that this promising technique is safe and effective. In the current review, we aim to provide background information on the anatomy, physiology, and pathophysiology of the ET and to present the progress of the balloon dilation technique with emphasis on pediatric patients.

## Introduction and background

The eustachian tube (ET) is not merely a canal that connects the tympanic cavity with the nasopharynx but is a distinct organ that plays a fundamental role in the physiology of the middle ear. Its first role is to aerate the middle ear, creating a condition of equal air pressure on both sides of the tympanic membrane. In this way, the tympano-ossicular system remains at the state of least impendence, transmitting, consequentially, the mechanical wave of sound to the inner-ear lymph in the most effective way [1,2]. An additional role includes the clearance of secretions, as the middle ear clears its mucus towards the nasopharynx with the aid of the ciliated respiratory epithelium of the ET. Simultaneously, the muscles surrounding the tube exert a pumping action from proximal to distal, propelling mucus to the nasopharynx [1,2]. A third role of the ET is the protection of the middle ear [1,2]. A manifestation of its protective role is the inhibition of pathogen-laden secretions and sounds created in the nasopharynx from entering the middle ear. The ET prevents pathogens from ascending to the ear by remaining mostly closed; it also features a mucosal surface coated with surfactant. The surfactant acts both as a surface-tension reducer and as an antibacterial substance [1]. Finally, the air cushion, according to the flask model of C. Bluestone, where the ET plays the role of the narrow bottleneck, constitutes an additional line of defense [1].

Eustachian tube anatomy was first described by the Italian anatomist Bartolomeo Eustachi in 1562. In 1704, Antonio Valsalva described the muscles that surround the tube. The ET in adults has a total length of 31-40 mm, with an average of 36 mm. Its tympanic orifice is located inferior and lateral to the promontory of the inner ear. The bony part of the ET with a length of 10-13 mm is part of the protympanum. There is a junctional part of 3 mm, where the cartilage is overlapped by bone. The narrowest part, the isthmus, is located approximately 3 mm distal to the junctional part. The diameter of the isthmus is about 1.5 mm. The cartilaginous part, also known as the valve area, has an average length of 28.6±2.5 mm, with minimum and maximum referred lengths of 22.5 mm and 36 mm, respectively. The pharyngeal orifice at the torus tubarius has a surface of 9x5 mm. The inclination of the ET to the horizontal level is 30°-40° and to the sagittal level is 45° in adults. The bony and the cartilaginous parts are not in line but form a 160° angle [2-4].

Eustachian tube dysfunction

The ET may be dysfunctional either because it is more obstructed or more patulous than normal. A third dysfunction category exists when the patient’s middle ear exhibits a condition of baro-challenge in situations of abrupt alterations of atmospheric or hydrostatic pressure on the tympanic membrane. This is a manifestation of a subclinical obstructive dysfunction [5]. The most common symptoms of patients suffering from a patulous eustachian tube (PET) are autophony, breath autophony, blocked ear sensation, and a feeling described as if they are in an empty barrel [6,7]. This condition is primarily idiopathic. Occasionally, it may be related to recent weight loss, hormonal disturbances, dehydration, radiotherapy, and neurological disorders [8]. Its differential diagnosis includes conditions like a dehiscent superior semicircular canal, temporomandibular joint dysfunction, and Meniere’s disease [6,7]. Very rarely would a child present with symptoms of a PTE. Presumably, the above-mentioned risk factors for PET are not common in the pediatric population. Operative procedures for the patulous dysfunction that aim to obstruct the ET lumen partially could not be acceptable therapeutic options for children. This fact is reflected in the paucity of bibliography on operative procedures for PET in children.

Obstructive dysfunction

An obstructive dysfunction of the ET can be either anatomical or functional. Several conditions can obstruct the tube anatomically. Some are located inside the lumen, like allergy, inflammation, and edema from gastroesophageal reflux. Others are located outside the lumen, like hypertrophic adenoids and neoplasia of the nasopharynx.

Before focusing on the interpretation of the functional obstructive dysfunction, it is necessary to describe the mechanism that normally opens the ET. On the one hand, an alteration in the atmospheric pressure between the nasopharynx and middle ear can passively open the tube, letting air enter or exit. On the other hand, an active opening mechanism is mediated by muscular attachment to the tube. The tensor veli palatini (TVP) is a muscle that ends at the palatal aponeurosis, and its role is to tense the soft palate. It arises from a flat lamella of the scaphoid fossa at the base of the medial pterygoid plate, from the spina angularis of the sphenoid, but also from the lateral wall of the cartilage of the ET. Every time the TVP muscle contracts during swallowing and yawning, the eustachian tube opens for approximately 500 ms, letting a bolus of air enter or exit. It is noteworthy that the eustachian tube opens actively every 1-2 minutes during swallowing [9]. However, not every swallow maneuver achieves opening the tube [9]. Interestingly, it has been shown that the levator palatini and salpingopharyngeus muscle have a minor role in the opening of the tube [4,10]. Thus, any pathology that affects either the TVP muscle itself or its innervations and the geometry of muscle attachment to the cartilaginous part of the tube may result in a functional obstructive dysfunction. Typically, the condition mentioned appears in children younger than seven years old due to immature cranial base development. A similar condition is met in patients who suffer from a cleft palate due to the unfavorable geometry of the muscles that attach to the soft palate [11].

Differences in eustachian tube between children and adults

Children are more vulnerable to middle ear pathology than adults. This is attributed chiefly to the fact that a dysfunctional ET is a common condition in children. The ET in children exhibits several differences compared to adults [1]. In children, the tube is shorter in total, but with an osseous part, relatively longer when compared to adults. Additionally, the ET in children is more horizontal. The inclination of the tube relative to the horizontal level in a neonate is approximately 10°. Moreover, in children, the osseous-cartilaginous junction appears inline. These differences change the geometry of the TVP muscle’s attachment to the tube’s cartilage, rendering the active opening of the tube; less effective in children. The histological composition also features differences, including more dense cartilage with less elastin for the ET in children. Additionally, Ostmann’s fat pad, located laterally to the lateral wall of the tube’s cartilage, is relatively more massive in children. Furthermore, the mucosa is thicker and more folded in children. Finally, the children’s tube submucosa is characterized by more developed lymphoid tissue aggregations that form the tubal tonsil. The same stands true for the more prominent adenoids in children than adults.

Additional parameters implicated in the eustachian tube dysfunction in children

Several parameters are implicated in the pathogenesis of ET dysfunction [1,12]. Viral infections, which are much more common during childhood, create both anatomical and functional obstruction. This occurs due to mucous production, mucosal edema, submucosal lymphoid tissue hyperplasia, and transient damage to the ciliated epithelium. The formation of a biofilm of pathogens preserves an inflammatory condition. Allergic rhinitis, as well as gastroesophageal reflux, can also contribute to ET dysfunction. The clearance function of the ET can also be disturbed because of - either primary or secondary - disorders of mucous and mucosa. Characteristic primary disorders are cystic fibrosis disease and cilia dysmotility disease, while secondary disorders are often induced by exposure to smoke [13]. Bottle feeding and pacifier usage, especially when breathing from the nose is obstructed, can create a Toynbee phenomenon that leads to negative pressure in the middle ear. Hereditary or racial anatomic parameters make individuals more vulnerable to ET dysfunction. This becomes clearer in specific pathologic conditions, like syndromes that are implicated with dysplasia of the cranial base, submucosal cleft palate, and neuromuscular dysfunction, which can influence the mechanisms of the active tube opening.

Eustachian tube dysfunction sequels

A chronic ET dysfunction, defined as a condition that lasts for more than three consecutive months, is a problem that affects 1-5% of the adult population [14]. On the contrary, almost 40% of children may face the consequences of ET dysfunction in the form of chronic or recurrent otitis media with effusion [1].

Effusion in the middle ear is an exudation that is formed under the condition of negative pressure that exceeds -100 mm H_2_O. This vacuum of air is induced when nitrogen, the gas with the largest diffusion gradient, is absorbed by micro-vascular circulation quicker than it can be replaced due to obstructive dysfunction of the ET. Chronic otitis media with effusion is a condition that renders children vulnerable to recurrent episodes of purulent otitis media, but also chronic conductive hearing loss (CHL). CHL during the sensitive period when children are developing their speech can be way more detrimental, mainly since, apart from speech delay, CHL can be related to central auditory processing disorders even years after the resolution of the effusion [15]. Moreover, chronic middle ear under-pressure can lead to retraction pocket formation in pars flaccida of the tympanic membrane [16]. This can lead to a later sequel that concludes with cholesteatoma formation. Moreover, the tympanic membrane may become atelectatic or symphytic. Finally, the development of cholesterol granuloma and tympanosclerosis can be expected [16].

Contemporary management of otitis media with effusion

The latest guidelines do not support the medical treatment for otitis media with effusion [17]. Medical approaches like antihistaminics, montelukast, proton pump inhibitors (PPIs), decongestants, and nebulized surfactants have been proven ineffective. Other approaches with antibiotics, systemic and local steroids, and auto-inflation devices do not achieve long-term effectiveness [17].

The criteria for surgical management are based on the chronic nature of effusion, the level of hearing loss, the frequency of episodes of purulent otitis media, and the retraction of the tympanic membrane. Insertion of tympanic membrane grommets that manage to aerate the middle ear externally is the first-line approach [17]. Adenoidectomy is an additional therapeutic option. Adenoidectomy involves removing a tissue that may mechanically obstruct the ET but which can also maintain an inflammatory condition due to the pathogens’ biofilm that it hosts. It is nevertheless considered an adjuvant operation for otitis media with effusion, and it is recommended only in children over four years old, irrespective of the adenoidal mass. In younger children, adenoidectomy is suggested only when additional criteria for obstructed sleep apnea are fulfilled [17]. Grommets are intended to aerate the middle ear for several months. Subsequently, when the tympanic membrane extrudes them, aeration’s beneficial results will hopefully be preserved since adenoids have been removed and the child’s ET has further developed.

It is noteworthy that even if the pathophysiology of the otitis media with effusion is based mainly on ET dysfunction, all the aforementioned surgical approaches indirectly influence the tube. More radical ET reconstructive approaches developed in the 1950s targeting the bony part and the middle ear ostium were deemed risky, with no benefit for the patients [18]. Thus, they never became an option in the therapeutic armamentarium for ET dysfunction.

## Review

Focusing on the eustachian tube

The new era for surgical approaches focused on ET rose in 1997 when Kujawski first attempted laser plastic reconstruction of the torus tubarius. The first publication on laser reconstruction came in 2003 by Poe et al. [19]. The rationale behind this technique is to open the distal part of the cartilaginous valve by laser cauterization of the medial lamina of the ET cartilage at a depth of about 3 mm. The cauterization included mucosa, submucosa, and cartilage. The authors concluded that this procedure is successful in almost 70% of the patients. However, despite several articles published on that method in the following years, interest had begun to wane by 2007. This year a new method was published by Metson et al., where plastic reconstruction of torus tubarius was performed by debrider [20]. Successful attempts were presented in a limited series of patients, but there have been no other references on that method. One additional reason for abandoning these operations was the evolution of a new method based on dilation of the cartilaginous part of the ET with a balloon. Similar interventions were not unfamiliar to otolaryngologists since balloon dilation had already been used to dilate the osteomeatal complex in functional endoscopic sinus surgery [21].

Balloon dilation of the cartilaginous part of the ET was first attempted in Bielefeld, Germany, by Ockermann in 2009. It was accompanied by two publications the following year, one on cadavers [22] and another on a series of eight patients [23]. Since then, a growing number of publications from many different countries and institutions, meta-analyses, and reviews have enriched the relevant bibliography.

Dilation technique

The technique is not too complicated; it lasts about 5-15 minutes and has a fast learning curve [24]. An angulated guiding catheter of 30°, 45°, or 70° is inserted through the ipsilateral nostril to the pharyngeal orifice of the ET. The operation is done under endoscopic vision by a rigid endoscope inserted either from the ipsilateral, the contralateral nostril, or even the mouth. Another catheter that bears a balloon is inserted through the guiding catheter. As the catheter is pushed through the cartilaginous part of the ET, the operator feels a mild resistance when the wire reaches the narrow isthmus part of the tube. Some balloons bear a colored indication for the depth of insertion. The balloon is inflated with saline to a pressure that ranges from 8 to 12 bar for one or two minutes [25]. Some articles support an additional round of dilation after 2 minutes [26]. An interesting cadaveric study indicates that plastic deformation of the cartilage begins at 5 bar and shows no further benefit over 10 bar. It is shown that ruptures on the cartilage can occur at pressures over 12 bar [27].

Available systems

Currently, three dilation systems are commercially available. The SPIGGLE and THEIS system from Germany was used since early German publications [28]. It features a balloon with dimensions of 3x20 mm, which becomes 3.39 mm when inflated [27]. Additionally, this system is reusable and translated at a relatively low cost. A disposable (hence more costly) system available from the USA is Acclarent AERA (Irvine, CA: Johnson & Johnson) [29]. There are different options for the balloon width from 5 to 7 mm, which increases by 0.58 mm when inflated [27]. The balloon comes in different lengths, varying from 15 to 24 mm. This system has been in use since the first attempts in the USA, but its usage was initially off-label. It finally received FDA approval for adults in 2016, but its usage is still off-label in children. A third system, a more rarely referenced system, is the XprESS LoProfile ENT dilation system (Plymouth, MN: Stryker ENT) from the USA [30]. It comes in dimensions of 5-7 mm in width and 8-20 mm in length.

Mechanism of action

Various hypotheses have been used to explain the mechanism of action of ET dilation [27]. Micro-ruptures on the cartilage that are healed by collagen type I lead to alterations in the architecture of the tissue making the tube less floppy. Additionally, the balloon can split connective tissue synechiae that are formed in the lumen of the tube after recurrent inflammations. Recent indications support the hypothesis that the pressure from the balloon crushes lymphatic tissue in the submucosa, leading to fibrosis which makes the tube more rigid. The crushed ciliated epithelium regenerates in the next six weeks. The new epithelium is considered to be healthier, coated with less pathogenic biofilm, and with sufficient amounts of surfactant. Finally, there is a theory based on the existence of mechanoreceptors on the tube whose stimulation by pressure may affect TVP muscle activity [31].

Evaluation of eustachian tube function

The majority of the original articles presenting ET dilation outcomes compare pre- and post-operative results of either simple physical or even more sophisticated tests [32,33]. Among the most frequent examinations is simple and pneumatic otoscopy, which assesses the existence of retraction and symphysis of the tympanic membrane. Tympanographic results are essential for evaluating improvement or restoration to a normal condition characterized by a tympanogram type A. An audiogram is also very important since the improvement of hearing loss is the main objective of our intervention. Many studies focus on restoring Valsalva and Toynbee maneuver, assessed otoscopically or tympanometrically. It is important, though, to stress that almost 20% of normal people cannot perform these maneuvers successfully.

More sophisticated equipment but not widely available, like pressure champers, may also be used to evaluate the response of ET function to alterations of atmospheric pressure. Examinations like tubomanometry and the nine-step test are useful for ET evaluation when the tympanic membrane is intact [34]. However, they are proven less practical for pediatric cases. Other examinations like the inflation-deflation test and forced response test evaluate only the passive opening of the tube in a non-intact tympanic membrane and abnormal conditions of pressure variations. Finally, sonotubometry is a very promising examination [35]. The main idea is to detect alteration in a transmitted sound at the external ear canal during the test. This is a sound artificially produced at the nasopharynx and transmitted to the middle ear when the patient is synchronously performing maneuvers that actively open the ET, like yawing. The main advantage of this technique is that it evaluates ET opening in physiological conditions avoiding the abrupt alteration of pressures.

Many investigators perform nasopharyngeal endoscopy for a differential diagnosis regarding ET obstruction. Additionally, evaluation of the level of inflammatory indexes of torus tubarius by a slow-motion video-endoscopy may be useful. Such indexes include mucosa hypertrophy, redness, cobblestoning, and mucus quality [36]. Other useful tools, mainly for pre- and post-operative comparison of symptoms and quality-of-life, but not for the diagnosis, are the patient-reported outcome measures questionnaires Eustachian Tube Dysfunction Questionnaire-7 (ETDQ-7) and Cambridge Eustachian Tube Dysfunction Assessment (CETDA) [37,38]. It is important to mention, though, that they are not able to discriminate between patients with obstructive and patulous ET dysfunction, that they demand an intact tympanic membrane, and that their reliability in children is rather questionable [38]. Many evaluations utilize ET scores in combination with other examinations. Such combined tests include, for example, a score that combines ETDQ-7, tympanometry, and click sounds created by ET opening during swallowing or yawning [25]. In other cases, a combination of examinations that includes Valsalva maneuver, tubomanometry in 30-40-50 mbar, plus click sound of the tube has been used [25]. Another ET score combines ETDQ-7, Valsalva maneuver, and tympanometry [23]. Few articles have also evaluated the clearance function of the ET from the middle ear to the nasopharynx using fluorescein dyes or radioactive substances [39].

Radiographic tests, like CT and MRI, though able to provide valuable information regarding ET dysfunction, are not part of the routine ET assessment [40]. However, the pre-operative evaluation with CT remains a point of debate. In early publications, it was pretty common to perform CT pre-operatively to avoid dilation in cases of aberrant carotids or in case of a dehiscent bony part of the tube to the carotid artery. Later publications, including consensus opinions of experts, supported that CT cannot predict such difficulties regarding aberrant anatomy that could influence the decision for the dilation of the ET [41]. Since the technique does not intend to dilate the bony part of the tube, the existence of a dehiscent bony wall is not very critical. Currently, CT is performed only in centers where in addition to the dilations, they proceed to adjuvant interventions, like a passage of the bony part by a flexible metallic wire [42]. On the other hand, CT can be proven a valuable aid in the differential diagnosis when otologic symptoms are somewhat vague, like in the case of a dehiscent front semicircular canal. The last condition is similar to ET dysfunction, characterized by symptoms like blocked ear sensation and autophony.

Results of eustachian tube dilation

Eustachian tube dilation is an advantageous therapeutic approach for obstructive and baro-challenge dysfunction. A growing number of original articles, reviews, and meta-analyses support this procedure’s efficacy, especially in adults [25,34,43-51]. This paragraph collectively presents the results of the studies mentioned above that indicate the positive impact of ET dilation on various parameters. The follow-up period in different series varies between eight weeks and three years. Interestingly maximum improvement occurs after the sixth week when the ciliated mucosa is expected to recover. Type B tympanogram is restored to a normal type A in 52-87% of cases. Valsalva maneuver can be performed pre-operatively in approximately 8% of patients, while the capability of performing the Valsalva maneuver varies between 70% and 100% post-operatively. A significant improvement on ETDQ-7 of more than 2.5 units occurs in 56% of cases of dilation compared to 8.5% of controls in six weeks. Finally, the scoring of mucosa inflammation indexes of torus tubarius improves in 72% of the patients that undergo dilation. It is noteworthy that dilation not only improves obstruction of the tube but also indirectly affects the active-opening muscular mechanism since the TVP muscle requires less effort to open the tube.

Complications

The main reason for the late development of ET dilation techniques was the fear of severe complications concerning the carotid artery (CA), which presents a close anatomical relationship with the bony part of the ET [22,52,53]. Moreover, the bone may even be dehiscent to the carotid in 7% of the population. Such anatomic variation would, theoretically, render the CA even more vulnerable to trauma during manipulation of the ET. Although early cadaveric studies were skeptical about CA safety, there is no case of complications from the CA in the international bibliography [41,54]. Another theoretical complication of dilation was the transition of an obstructive dysfunction of the ET to a patulous dysfunction after the procedure. Again, there are no references for such a complication. The most frequent complication is epistaxis met in 3% of the operations controlled with conservative measures [55]. Hemotympanum, which resolves without any intervention, is another possible complication. Subcutaneous emphysema has been described in 1% of the procedures [56]. This may happen either due to gross cartilaginous rupture or due to false passage in the process of propelling the wire with the balloon. It usually resolves in 48 h, but antibiotics and abstinence from the Valsalva maneuver for three weeks should be recommended [56]. Finally, there are sparse references to sensorineural hearing loss after dilation which can be attributed to rupture of the round window due to barotrauma during dilation [57]. The alterations in middle ear pressure during the dilation procedure and the potential of barotrauma had been initially a concern that led some investigators to suggest myringotomy before the dilation. However, it has been shown both in cadaveric [58] and in patient [59] studies that dilation induces middle ear pressures within the normal physiologic range.

Results of eustachian tube dilation in pediatric series

Pioneer centers that developed the dilation technique also made early attempts on pediatric patients. The first publication of a pediatric series came from Ulm, Germany, in 2013 [60]. The bibliography that concerns children is not as rich as that of adults, primarily due to the lack of FDA approval for dilation in children. However, some well-organized original studies and meta-analyses have already been published [26,32,34,55,61-65]. Most pediatric series use the SPIGGLE and THEIS dilation system (TubaVent; Overath, Germany: SPIGGLE & THEIS Medizintechnik GmbH), but some publications use the Acclarent AERA 6x16 mm and Acclarent 3.5x10 mm, even if they are not FDA approved yet. In most publications, the median ages of patients varied between seven and 12 years old. Notably, in a seven years old child, the average length of the cartilaginous part of the ET is approximately 24 mm, which is 84% of the average length of the cartilaginous part of an adult. Contrary to adults, dilation is not used as a first-choice therapeutic approach in pediatric series. The authors resorted to ET dilation only after unsuccessful attempts with adenoidectomy and grommets. However, other pediatric series support the advantageous efficacy of the ballon tuboplasty even as a first-choice therapeutic approach [66], as well as the beneficial combination of ET dilation with grommets, especially regarding the long-term results [67]. Moreover, the dilation protocol in pediatric series uses either 8, 10, or 12 bar pressure for 1 or 2 minutes. It is encouraging that there are no references of any severe complications apart from epistaxis and hemotympanum in the pediatric series.

A recent indicative publication from Toivonen et al. [65] provides a detailed description of ET dilation using Acclarent 6x16 mm [65]. The balloon pressure was set at 12 atm for 1 or 2 minutes. The duration of the dilation was based on the degree of the endoscopic inflammatory indexes of the torus tubarius. The series included 27 patients whose median age was 12.5 years. The children enrolled in the study should have had ET obstructive dysfunction symptoms for at least nine years and should have undergone multiple operations for grommet insertion or adenoidectomy. Additionally, they should have undergone an unsuccessful therapeutic attempt with cortisone spray and PPIs for at least six weeks before dilation. The authors did not report any complications. The follow-up was scheduled for six, 12, and 36 months after the procedure. None of the children had normal otoscopy before dilation, while at six, 12, and 36 months 38%, 55%, and 93% of children had a normal tympanic membrane, respectively. Moreover, none of the children had a normal A tympanogram before dilation, while in the three consecutive follow-up examinations, tympanogram A was found in 50%, 59%, and 85% of children, respectively. Air/bone gap in audiogram was 18 dB before the procedure with gradual narrowing in the consecutive follow-up examintions, up to 6 dB in 36 months. The scoring of inflammatory signs of the torus tubarius’ mucosa gradually decreased after the dilation, and the capability to perform the Valsalva maneuver improved. An interesting fact is that comparing dilation and grommets insertion showed that two years after each of the two invasive procedures, the non-recurrence probability of otitis media with effusion was 87% for dilation cases and 56% for grommets.

Novel and experimental dilation approaches

Eustachian tube dilation is a new technique that will undoubtedly evolve with time. Novel approaches include navigation-assisted dilation [68] and dilation under fluoroscopic guidance [69], developed in certain Korean institutes. Such techniques may assist safety; however, unacceptable radiation exposure renders the value of those procedures questionable. Additionally, parallel with dilation of the cartilaginous part of the tube, adjuvant procedures like an exploration of the bony part of the tube with an illuminated guidewire of 0.9 mm in an attempt to assist lysis of mucosal adhesions may be performed [42]. The efficacy of dilation of the cartilaginous part of the tube through the transtympanic approach has also been tested [70]. However, safety concerns have emerged, as well as concerns regarding the impracticality of such an intervention [71]. One promising experimental technique that could assist diagnosis, but also anatomical and pathophysiological studies, is optical coherence tomography (OCT) with a specialized round-tip catheter in the ET [72]. Moreover, cadaveric studies have shown the efficacy of standard endovascular balloons for ET dilation [73]. Finally, there have been some interesting experimental techniques in cadavers with metallic stents in the cartilaginous part of the tube [74] and with tensor veli palatinopexy in an attempt to improve the muscular mechanism of active tube opening [75]. Nevertheless, ET dilation is a promising technique that will undoubtedly evolve and eventually gain popularity in the future; it has already been proposed that ET dilation may be efficiently performed under local anesthesia in an office setting, substantially reducing costs [76].

## Conclusions

Eustachian tube obstructive dysfunction is a common issue, especially in the pediatric population. It constitutes a significant burden on the quality of life, leading to hearing loss and making children prone to otitis media and cholesteatoma formation. Current therapeutic approaches with grommets and adenoidectomy target the consequences of the dysfunctional tube only indirectly. Dilation with balloons is a novel, safe, and effective technique, both for adults and children, which directly addresses the underlying pathology of obstructive ET. Promising results from more extensive pediatric series and approval of the technique from food and drug administration agencies are anticipated to allow for further familiarization of ENT surgeons with this intervention and thus, enrich the therapeutic armamentarium against ET obstructive dysfunction.
